# Disease-associated metabolic alterations that impact satellite cells and muscle regeneration: perspectives and therapeutic outlook

**DOI:** 10.1186/s12986-021-00565-0

**Published:** 2021-03-25

**Authors:** Josiane Joseph, Jason D. Doles

**Affiliations:** 1 Mayo Clinic Medical Scientist Training Program, Mayo Clinic, Rochester, MN USA; 2 Department of Biochemistry and Molecular Biology, Mayo Clinic, 200 First St SW, Rochester, MN 55905 USA

**Keywords:** Atrophy, Degeneration, Metabolism, Muscle wasting, Myoblasts, Satellite cells

## Abstract

Many chronic disease patients experience a concurrent loss of lean muscle mass. Skeletal muscle is a dynamic tissue maintained by continuous protein turnover and progenitor cell activity. Muscle stem cells, or satellite cells, differentiate (by a process called myogenesis) and fuse to repair and regenerate muscle. During myogenesis, satellite cells undergo extensive metabolic alterations; therefore, pathologies characterized by metabolic derangements have the potential to impair myogenesis, and consequently exacerbate skeletal muscle wasting. How disease-associated metabolic disruptions in satellite cells might be contributing to wasting is an important question that is largely neglected. With this review we highlight the impact of various metabolic disruptions in disease on myogenesis and skeletal muscle regeneration. We also discuss metabolic therapies with the potential to improve myogenesis, skeletal muscle regeneration, and ultimately muscle mass.

## Introduction

Skeletal muscle is a vital organ that supports locomotion, respiration, vision, and posture. Additionally, skeletal muscle significantly contributes to organismal physiology through energy production and endocrine signaling [1, 2]. To accommodate these and other daily activities, skeletal muscle cells can increase or decrease mitochondrial biomass [3]. Adjustments to protein content and enzymatic activity in muscle also supports normal function. As a consequence of many chronic diseases—such as Huntington’s Disease [4], chronic kidney disease [5], various cancers [6, 7], lung pathologies [8], and myopathies—abnormal shifts in skeletal muscle metabolism frequently arise.

In addition to disruptions in cellular metabolism, many chronic diseases are often associated with significant skeletal muscle wasting. Muscle wasting results in unfavorable loss of functional capacity and quality of life in affected individuals. Despite substantial basic and clinical work to understand and address this prevalent issue, there are no consistently successful therapies to resolve muscle wasting. These limitations suggest that our current approach towards the issue of muscle wasting should be refocused. Skeletal muscle mass is maintained by a balance of protein anabolism, protein catabolism, and cellular turnover. Though muscle atrophy is primarily attributed to increased protein degradation without compensated protein production [9, 10] , some recent evidence suggests that impaired regeneration may also play a role [11]. Muscle repair involves necrosis of injured tissues, invasion of leukocytes, clearance by phagocytic cells, scarring, revascularization, fusion of muscle progenitor cells, and remodeling to restore functional capacity of the muscle [12]. Indeed, a diverse community of cells is required to regenerate muscle after it is damaged by normal use or injury.

The functional adult muscle progenitor cell is the satellite cell. These cells are located between the sarcolemma and basement membrane of muscle fibers and are frequently adjacent to blood vessels [13, 14]. Satellite cells are stimulated under stress to proliferate, differentiate, and fuse into muscle fibers by a process called myogenesis [15]. In addition to contributing new nuclei and cellular material to terminally differentiated myofibers, satellite cells must replenish the quiescent reserve of satellite cells for subsequent muscle repair, in a process termed self-renewal. With all of the events that must occur in sequence in order for skeletal muscle to successfully regenerate, there are multiple avenues for pathological disruption.

While quiescent, satellite cells primarily utilize fatty acid and pyruvate oxidation to meet energetic demands but switch to aerobic glycolysis when activated [16]. After activation satellite cells divide asymmetrically. This expansion is synced with mitochondrial biogenesis [17]. As they differentiate, myoblasts (or activated satellite cells) increase mitochondrial biomass in favor of oxidative phosphorylation [18, 19]. To accommodate the metabolic adjustments that occur during each phase of myogenesis, functional autophagy programs are required [20]. Thus, factors that impair autophagy have the potential to undermine skeletal muscle repair. With this review we highlight several disease-associated metabolic disruptions that alter satellite cell function and impact skeletal muscle regeneration and emphasize potential avenues for future investigation. Additionally, we discuss metabolic interventions reported to boost satellite cell function and/or skeletal muscle regeneration.

## Metabolic disruptions in disease that impact skeletal muscle regeneration

Skeletal muscle is a highly metabolic tissue and muscle regeneration capacity is dependent on multiple variables. For example, satellite cell quantity, activation and proliferation, myogenesis, and fusion all contribute to skeletal muscle regeneration. Pathologies that directly alter muscle metabolic activity or that systemically affect cellular metabolism—such as obesity, diabetes, aging, neuromuscular disorders, and sepsis—influence the ability of satellite cells to function in their primary role of regenerating muscle (Table 1).

**Table 1:** Pathologies associated with altered cellular metabolism and satellite cell dysfunction

Investigators	Model	Metabolic disturbance(s)	Outcome
Tamilarasan et al*.* [11]	MCK(m)-hLPL mice	Striated muscle targeted elevation of lipids	Reduced muscle weight and cross-sectional area (or regeneration) after injury
Tamilarasan et al*.* [11]	hLPL transfected C2C12 myoblasts	Elevated intracellular FFA	Impaired myoblasts fusion and myogenesis
Fu et al*.* [86]	High-fat diet fed mice	Obesity	Reduced muscle regeneration and reduced primary satellite cell pool
D’Souza et al*.* [25]	C57BL/6-Ins2^Akita/J^ mice	Type I diabetes	Reduced muscle weight Reduced satellite cell activation and proliferation Reduced myogenic transcripts
Xu et al*.* [26]	16 week High-Fat Diet fed mice	Pre-diabetes/Obesity	Impaired primary satellite cell differentiation
García-Prat et al*.* [36]	GFP–LC3 Geriatric mice	Advanced age/Senescence	Impaired satellite cell autophagy Impaired expansion and fusion of transplanted myoblasts
García-Prat et al*.* [36]	Human primary myoblasts	Advanced age/Senescence	Impaired clearance of cellular components and increased ROS Reduction in proliferation
Servián-Morilla et al*.* [37]	Limb-girdle muscular dystrophy 2H patients	Premature senescence Fatty replacement of muscle	Reduced myoblast proliferation and differentiation Increased autophagic flux in myoblast
Kudryashova et al*.* [38]	Trim32^–/–^ (Limb-girdle dystrophy) mice	Premature senescence	Myoblast growth arrest Impaired myoblasts self-renewal and differentiation
Yablonka-Reuveni et al Bell et al	mdx mice	Mitochondrial abnormalities [47]	Accelerated muscle regeneration [42]
Schaaf et al*.* [48]	Pompe patients (4 disease groups)	Lysosomal glycogen accumulation	Impaired satellite cell activation and differentiation
Lagalice et al*.* [49]	GAA-KO 6^neo^/6^neo^ (Pompe) mice	Lysosomal glycogen accumulation Lipid accumulation	Impaired muscle regeneration Increase satellite cell activation (and quantity for young mice only)
Schaaf et al. [50]	*Gaa*^−/−^ (Pompe) mice	Lysosomal glycogen accumulation	Increased satellite cell pool Impaired muscle regeneration
Manzano et al*.* [55]	SOD1-G93A mutant (ALS) mice	Altered superoxide dismutase function	Alterations in satellite cell abundance and activation
Gouzi et al*.* [8]	COPD patients	Elevated ROS	Increased myoblast autophagy
Pomiès et al*.* [63]	COPD patients	Increased oxidative stress	Myoblasts with: greater susceptibility to oxidative stress, reduced fusion capacity, increased protein carbonylation Myotubes with reduced diameter
Rocheteau et al*.* [64]	Cecal ligation and puncture in mice	Sepsis	Impaired satellite cell proliferation Reduced satellite cell quantity Impaired muscle regeneration Altered satellite cell mitochondria

### Obesity

In the context of obesity, adipose tissue accumulates in muscle where it can secrete compounds that influence neighboring cells. Lipids are known modulators of satellite cell function [21, 22]. Lipoprotein lipase (LPL) aids in lipid catabolism for cellular uptake [23] and these hydrophobic molecules may accumulate and alter cell function. For example, lipid overload (cause by LPL overexpression) can inhibit early myogenesis, impair satellite cell proliferation, and reduce regeneration capacity [11]. The overall depletion of lean mass observed in obese individuals [24] may partially be attributable to ineffective satellite cell activity. In line with this prediction, MCK(m)-hLPL mice with elevated lipids, specifically in striated muscle, have reduced type II muscle weights when compared to wildtype controls [11]. Inflammation and insulin resistance are also traits of obesity that may negatively influence muscle mass; however, that does not negate the potential role of satellite cell metabolic dysfunction in muscle wasting.

### Diabetes

Though the pathogenesis of type I and type II diabetes is distinct, they are similarly characterized by elevated blood glucose. Interestingly, individuals with type I diabetes mellitus have fewer satellite cells than healthy controls [25]. Additionally, satellite cells from a type I diabetic environment exhibit impaired activation and self-renewal capacity [25]. Again these differences were observed concurrently with a reduction in muscle weight suggesting an association [25]. While the former study did not assay regeneration, another group recently determined that myogenesis is hindered in mice with impaired fasting glucose and glucose tolerance [26]. Though multiple studies have identified muscle wasting in diabetic models, the cause of the muscle loss is uncertain [27, 28]; what is becoming clear is that satellite cells are defective in this disease.

### Aging

As age increases, tissue regenerative capacity decreases. There are physiological changes that occur with age—such as declines in sex hormones—that are related to reduced muscle mass and modified cellular compartments. Several investigators reported that satellite cell quantity [29] and function remains fairly constant with age [30], while others showed age-associated satellite cell depletion [31] and diminished self-renewal capacity [32]. Despite this discordance, what is clear is that aged satellite cells have altered metabolic profiles and likely use alternate carbon sources for energy production [33]. Additionally, glycolytic enzymes have an increased incidence of oxidation in senescent human myoblasts [33] which may play a role in the impaired regeneration and function of muscle observed in geriatric cohorts [34, 35]. Furthermore, autophagy function is frequently defective which begets a loss of satellite cell stemness in aged individuals [36]. As autophagy is also required for mitochondrial turnover (a process pertinent to satellite cell differentiation) [20] more effort should be made to decipher if the loss of skeletal muscle regeneration observed in aged individuals could be prescribed, to some degree, to satellite cell metabolic disruption.

### Myopathies and neuromuscular disorders

Skeletal muscle wasting occurs in metabolic disorders (such as Pompe disease) or may arise as a consequence of other factors that lead to altered metabolism in muscle. For example ALS is considered to be a neuronal pathology, however, as the disease progresses skeletal muscle wasting and metabolic defects are noted. Though the mechanisms by which neighboring tissues or pathologies achieve metabolic disruptions in muscle are unclear, there is abundant evidence that satellite cells are altered and regeneration may be impaired in a multitude of diseases.

As in aging, senescent satellite cells with dysfunctional metabolism are typical of a limb-girdle myopathy caused by mutated TRIM32. In this myopathy, myogenesis is impaired and overall there are fewer activated muscle stem cells [37, 38]. Satellite cells from TRIM32 mutated limb-girdle myopathy patients are characterized by abnormal autophagy activity and an accumulation of TRIM32 substrates [37]. TRIM32 regulates p62 activity and p62 targets other proteins for selective autophagy [39]. This disruption in autophagy may have implications for myogenesis and there is opportunity to understand how satellite cells may contribute to muscle wasting in individuals with this form of myopathy.

Dystrophin, the gene mutated in Duchenne muscular dystrophy, is substantially expressed in satellite cells [40]. Loss of dystrophin in satellite cells results in defective asymmetric cellular divisions and increased activation and is also associated with impaired muscle regeneration [40, 41]. Interestingly, despite the gradual loss of skeletal muscle mass in muscular dystrophy, dystrophic myoblasts exhibit accelerated differentiation [42]. This hyperactivity may contribute to the exhaustion of satellite cells which is suspected to lead to the severe deterioration of muscle [43, 44]. Although dystrophic satellite cells are defective they retain their ability to regenerate muscle fibers when transplanted to a wildtype host [45], indicating that the metabolically altered disease environment is a significant contributor to regenerative dysfunction. Dystrophic satellite cells are known to have distinct metabolic profiles when compared to cells from healthy controls [46]. Satellite cells from dystrophic mice also have fewer mitochondria, produce less ATP, are less susceptible to ATP synthase inhibition, and have reduced oxygen consumption rates [47]. Various metabolic defects in dystrophic satellite cells may play a role in the progressive muscle loss observed in Duchenne muscular dystrophy.

Pompe disease is caused by acid a-glucosidase deficiency. The resulting accumulation of glycogen in lysosomes causes loss of striation and vacuolization of muscle along with infiltration of leukocytes. The process of autophagy depends on functional lysosomes and as observed above satellite cells utilize autophagy for progression of myogenesis. Though the quantity and expansion of satellite cells in patients with Pompe disease is similar to healthy controls, satellite cell activation and muscle regeneration is impaired [48]. In mice, similar findings were reported with the additional observation of larger pools of satellite cells present in the muscle of younger mice [49, 50]. The impaired autophagy characteristic of lysosomal storage diseases [51–53] and reduced cross-sectional area of muscle myofibers in animal models of Pompe disease [49, 50] further supports the premise that satellite cell dysfunction may contribute to muscle wasting in this disorder.

A common cause of familial amyotrophic lateral sclerosis (ALS) is the mutation of a superoxide dismutase. Though the pathophysiology of ALS is unclear, skeletal muscle metabolic irregularities are suspected to contribute [54]. In this disease, satellite cells have variable abundance and capacity for activation depending on disease stage [55]. Additionally, satellite cells from ALS patients have morphologic features indicative of senescent cells and express senescence markers [56]. As previously discussed, senescent satellite cells have defective autophagy programs that preclude effective myogenesis. Altogether these data support our need to understand how skeletal muscle regeneration failure could be implicated in the muscle loss typical of this disease.

### Chronic obstructive pulmonary disease

For COPD patients, multiple variables may contribute to the skeletal muscle dysfunction often associated with disease including age, obesity, level of physical activity, prescribed steroids, tobacco use, systemic inflammation and reactive oxygen species [57]. Regulated shifts in reactive oxygen species (ROS) are associated with mitochondrial biogenesis and myogenic differentiation [58–61]. Myoblasts from patients with COPD appear to have increased autophagy accompanied by elevated reactive oxygen species [8]. These findings are consistent with other reports of satellite cells from COPD patients bearing features of senescent cells [62]. Though proliferation is unaffected in myoblasts from COPD patients, these progenitor cells are more susceptible to oxidative stress and have reduced fusion capacity [63]. The satellite cell deficits documented may contribute to the muscle atrophy noted in COPD patients.

### Sepsis

Sepsis physiology is characterized by impaired perfusion, inflammation, and ROS. All of these factors may play a role in the skeletal muscle wasting that is common among sepsis patients. Notably, there are fewer satellite cells with reduced capacity to regenerate muscle in individuals that have experienced sepsis [64]. Additionally, satellite cells in mice that have survived severe sepsis have reduced ROS and increased markers of oxidative stress (such as carbonylated and nitrated proteins) [64]. The alterations noted in satellite may lead to chronically impaired regeneration and futures efforts should be made to determine if they account for lasting muscle deficiencies observed in septic patients.

## Metabolic interventions that improve skeletal muscle pathology or promote muscle regeneration

As highlighted above, various disease processes coincide with dysfunctional skeletal muscle metabolism and impaired satellite cell function. As such, skeletal muscle metabolism may be an attractive target to mitigate muscle wasting, rescue impaired regeneration, and restore muscle function. Below, we highlight recent efforts to target muscle metabolism, including nutritional or small molecule supplements, hormone replacement, reduction of ROS, behavioral modifications, dietary modifications, and other therapeutic modalities. Effects on satellite cell function and/or muscle regeneration are also discussed.

### Select nutritional and small molecule interventions

Several common compounds are linked to improved muscle regeneration in diverse disease environments. A first example is acetoacetate which is a ketone body that improves skeletal muscle function and integrity in Duchenne muscle dystrophy [65]. In the dystrophic context, acetoacetate promotes muscle regeneration while improving satellite cell proliferation and differentiation [65]. Second, a flax seed rich diet also maintains muscle fiber integrity and improves regeneration in a model of dystrophic skeletal muscle [66]. A compound abundant in flaxseeds, alpha-linolenic acid, improves regeneration and reduces apoptosis of myogenic cells in vitro [66]*.* A third ubiquitous compound that may have regenerative benefits is nicotine. Nicotine promotes myogenesis in vitro and improves skeletal muscle regeneration in the context of obesity [67].

Other compounds that are easily purchased or commonly prescribed might be candidates to reverse muscle wasting. For example, geranylgeraniol, a molecule found in grains, fruits, and vegetables promotes myoblast differentiation and reduces the expression of proteins associated with atrophy [68]. Vitamin D (in the form of 1α-dihydroxyvitamin D3) improves human myoblast migration in vitro and enhances myogenesis [69]. Finally, rapamycin restores autophagy in satellite cells allowing for the clearance of cytosolic components and bioenergetics transitions [36].

Reactive oxygen species are required for normal cellular function but are frequently elevated in disease. Following this logic, antioxidants such as resveratrol, ursolic acid, and ascorbic acid are commonly evaluated for their therapeutic benefits. Treating myoblasts with resveratrol results in reduced apoptosis and improved differentiation in the presence of excess reactive oxygen species [70]. Seven day treatment with ursolic acid increases satellite cell quantity and fusion into myofibers [71]. Ascorbic acid increases myofibers diameter and reduces autophagy related mechanisms in cultured patient myoblasts [8]. Individuals with mitochondrial myopathies may require reactive oxygen species signaling for optimal muscle regeneration [72]. Although current interventions often favor restriction of reactive oxygen species, for certain myopathies, loss of reactive oxygen species could exacerbate pathology. Nevertheless, therapeutic targeting of reactive oxygen species remains promising because it can be accomplished by various means including non-invasive strategies such as nutritional modification and physical activity.

### Hormone replacement

Hormonal changes naturally occur with age. Growth hormone is typically reduced in subjects with advanced age, and loss of this hormone contributes to loss of muscle mass in geriatric individuals. Replacement of growth hormones improve mitochondrial biogenesis, reduce oxidative damage, increase protein synthesis, and increase factors associated with regeneration in muscle [73]. Unfortunately, growth hormone therapy is administered by regular injections, and there is no established consensus on its effectiveness at improving muscle function in humans [74, 75]. Despite the limitations, it may be worth exploring hormonal therapies for chronic and debilitating muscular disorders.

To combat age-related declines in sex hormone levels, replacement of testosterone for men and estrogen for women might also be considered to improve muscle mass. For example, satellite cell activation and muscle regeneration is facilitated by estrogen analog injection [76]. Moreover, testosterone increases satellite cell quantity and myonuclei, and is associated with muscle hypertrophy [77]. As with many hormonal therapies, however, there are systemic side-effects to consider with either of these options.

### Behavioral and hyperbaric oxygen interventions

Certain activities, such as weight training, are known to improve muscle mass. In disease, however, strenuous physical activity may not be possible. Alternatives or supplements to exercise become invaluable in such a scenario. Short-term caloric restriction increases satellite cell quantity, mitochondrial content, and proliferation and altogether contributes to muscle regeneration [78]. Improvements in satellite cell function were concurrent with an increase in oxygen consumption rate and a decrease in extracellular acidification rate [78]. Though caloric restriction appears to be beneficial in some models of myogenesis, the evidence varies with sex and by model and therefore requires further investigation [79].

Another proposed treatment option is hyperbaric oxygen immersion and/or inhalation. The treatment would entail breathing or immersing the body in oxygen at higher pressure in order to increase the oxygen content of tissues. Hyperbaric oxygen is already in use for non-healing wounds, decompression illness, and other indications. It is not standard therapy for muscle injury, but treatment with hyperbaric oxygen is an option to enhance muscle regeneration [80]. Hyperbaric oxygen increases satellite cell proliferation and differentiation while also facilitating macrophage invasion in damaged tissue which may explain the improvements in muscle regeneration and function observed after its use in rats [81]. Hyperbaric oxygen therapy also promotes muscle repair after injury by stimulating angiogenesis [82]. It is not clear if hyperbaric oxygen therapy can improve outcomes for chronic diseases. In Duchenne’s muscular dystrophy, for example, this treatment may not be beneficial [83].

### Biologics

Mesenchymal stem cell therapies are increasing in popularity and generally regulate inflammatory environments by secreting cytokines. In accord with known anti-inflammatory properties, intramuscular injection of mesenchymal stem cells reduces cytokine levels in septic mice [64]. Additionally transplanted mesenchymal stem cells improve muscle regeneration and restore mitochondrial activity in satellite cells of septic mice [64]. Intraperitoneal injections of bone marrow derived mesenchymal stem cells improves the morphology of myofibers, increase satellite cell quantity, and improves lifespan overall for Duchenne muscular dystrophy mice [84]. Two growth factors secreted by mesenchymal stem cells, CXCL12 and osteopontin, facilitate improved regeneration in dystrophic mice [84]. Despite a growing list of promising basic and preclinical mesenchymal stem cell studies, key obstacles remain. For example, stem cell-based therapies are challenging to standardize and repeated injections of a bulky and dynamic cellular product is not always favorable. Alternatively, injecting exosomes from mesenchymal stem cells may be an option. Indeed, intramuscular exosome treatment reportedly promotes angiogenesis, myogenesis, and muscle regeneration [85].

## Conclusions

In general, satellite cells are often overlooked when evaluating disease associated muscle wasting which may hamper our understanding of this syndrome. One limitation of many studies that assess metabolic dysfunction and wasting of muscle includes the focus on a single muscle, typically the tibialis anterior (a hind limb muscle), and neglect of other skeletal muscle types. Another barrier is the multitude of models designed to recapitulate the multifactorial muscle wasting syndrome hampers our ability to elucidate the causes (particularly related to altered metabolism) and effective treatments. This descriptive review outlines various metabolic abnormalities in satellite cells that are associated with wasting of skeletal muscle; due to the significance of metabolic regulation in satellite cell function our work is a start to interrogating how these metabolic disruptions may be enhancing refractory muscle wasting. There are options available to treat metabolic abnormalities in satellite cells and skeletal disease as detailed in this work. Considering the lack of effective treatment in the clinical setting, an improved understanding of disease-associated metabolic changes in satellite cells and how they impact muscle regeneration represents a promising path forward to addressing pathological loss of lean mass.

## Data Availability

Not applicable.

## Abbreviations

LPL: Lipoprotein lipase
ROS: Reactive oxygen species
